# An efficient and cost-effective method for disrupting genes in RAW264.7 macrophages using CRISPR-Cas9

**DOI:** 10.1371/journal.pone.0299513

**Published:** 2024-03-14

**Authors:** Mohammad J. Hossain, Tamara J. O’Connor

**Affiliations:** Department of Biological Chemistry, Johns Hopkins University School of Medicine, Baltimore, MD, United States of America; Hirosaki University Graduate School of Medicine, JAPAN

## Abstract

The clustered regularly interspaced short palindromic repeats (CRISPR) and CRISPR-associated protein 9 (Cas9) are widely used for genome editing in cultured cell lines. However, the implementation of genome editing is still challenging due to the complex and often costly multi-step process associated with this technique. Moreover, the efficiency of genome editing varies across cell types, often limiting utility. Herein, we describe pCRISPR-EASY, a vector for simplified cloning of single guide RNAs (sgRNAs) and its simultaneous introduction with CRISPR-Cas9 into cultured cells using a non-viral delivery system. We outline a comprehensive, step-by-step protocol for genome editing in RAW264.7 macrophages, a mouse macrophage cell line widely used in biomedical research for which genome editing using CRISPR-Cas9 has been restricted to lentiviral or expensive commercial reagents. This provides an economical, highly efficient and reliable method for genome editing that can easily be adapted for use in other systems.

## Introduction

The ability to edit eukaryotic genomes provides a powerful tool in studying cell biological processes. Several techniques have been developed to modify eukaryotic genomes including zinc finger nucleases (ZFNs) [1–3], transcription activator-like effector nucleases (TALENs) [4] and LAGLIDADG homing endonucleases [5]. The most recent addition to this toolbox is CRISPR-Cas9 [6,7], praised for both its simplicity and versatility [6–18].

CRISPR-Cas9-mediated genome modifications require the single guide RNA (sgRNA) and Cas9 be simultaneously introduced into the target cell. The most common method used involves packaging sgRNA and Cas9 encoding DNA into viral particles that are then used to infect mammalian cells [19]. While a highly efficient delivery system, generating virus particles is labor-intensive, time-consuming and costly [20]. The most frequently used alternatives to viral delivery systems rely on synthetic guide RNA or DNA and purified Cas9 protein that are introduced into cells by transfection, however these are typically less efficient and equally labor-intensive, time-consuming and costly [21–23].

Here we describe a single plasmid delivery system for CRISPR-Cas9 genome editing and a step-by-step protocol that streamlines screening for a robust, highly efficient and cost-effective method for genome editing in RAW264.7 cells. We have established a plasmid harboring Cas9 into which 20 base-pair oligonucleotides can be readily cloned to generate an accompanying target gene specific sgRNA, and does not rely on viral packaging to be introduced into target cells. To simplify the screening of CRISPR-Cas9 modified monoclonal cell lines, we have implemented a single step genomic DNA extraction method combined with target site PCR sequence analysis using the ICE tool [24] that circumvents the need for the widely used T7 endonuclease I (T7EI) and Surveyor endonucleases assays. To demonstrate its utility, we have applied this strategy to rapidly mutate genes in RAW264.7 cells [25], a mouse macrophage cell line widely used in biomedical research [26] for which genome editing using CRISPR-Cas9 has historically been achieved using lentiviral or costly commercial reagents [22,27–29]. The genome modification protocol described herein provides a detailed, economical guide to CRISPR-Cas9-mediated genome editing in mammalian macrophages that can easily be adapted for use in other cell types.

## Materials and methods

### Bacterial and RAW264.7 cell culture conditions

*E*. *coli* strains were cultured in LB medium supplemented with 50 μg/ml carbenicillin or 100 μg/ml ampicillin when appropriate. RAW264.7 cells (ATCC TIB-71) were cultured and maintained according to the recommendations by American Type Culture Collection (ATCC) in Dulbecco’s Modified Eagle Medium (DMEM) containing 10% fetal bovine serum at 37°C in the presence of 5% carbon dioxide, and supplemented with 10 mg/ml blasticidin when appropriate.

### Construction of sgRNA and CRISPR-Cas9 delivery plasmid

To introduce an sgRNA scaffold into pEF6-nls-YFP-2A-Cas9 [30], this plasmid was digested with *Aat*II and re-ligated to generate pYFP:Cas9v1 (pTO1406). To allow cloning of sgRNA-encoding oligonucleotides into the sgRNA scaffold using *Bsm*BI, the two *Bsm*BI recognition sequences in the *cas9* gene were mutated. Briefly, the *cas9 Bsm*BI fragment was amplified from pEF6-nls-YFP-2A-Cas9 using oligonucleotides EF63xnlsYFP2aCas9F and EF63xnlsYFP2aCas9R and cloned into *Bsm*BI digestpYFP-Cas9v1 using Seamless Ligation Cloning Extract (SLiCE) cloning [31] to generate pYFP:Cas9v2 (pTO1417). The sgRNA scaffold-encoding sequence was amplified by PCR in two pieces from LentiCRISPRv2 (Addgene plasmid #52961) [32] using oligonucleotides sgRNAscaffold-F1 and sgRNAscaffold-R1, and sgRNAscaffold-F2 and sgRNAscaffold-R2, respectively. The two fragments were then combined using splicing by overlap extension (SOE) PCR [33] using sgRNAscaffold-F1 and sgRNAscaffold-R2 and cloned into *Aat*II digested pYFP-Cas9v2 using SLiCE cloning to generate pCRISPR-EASY (pTO1463). All plasmids were confirmed by sequencing. All oligonucleotides used in this study are provided in S1 Table, and all plasmids in S2 Table.

### Designing gene-specific oligonucleotides for sgRNAs

For CRISPR-Cas9 genome editing of target genes, sgRNA specific oligonucleotides were designed using the GPP web portal developed by the Broad Institute (https://portals.broadinstitute.org/gpp/public/analysis-tools/sgrna-design). For genome editing in RAW264.7 cells, the mouse genome GRCm38 (NCBI RefSeq v.108) was selected as the Target Genome and the *Streptococcus pyogenes* Cas9 (SpyoCas9) and NGG were selected as CRISPR-Cas9 enzyme and protospacer adjacent motif (PAM), respectively. The top three sgRNA sequences (designated as ‘Pick Order’ 1, 2 and 3) were selected for each gene. To generate *Bsm*BI compatible cohesive ends for cloning into *Bsm*BI digested pCRISPR-EASY, the sequence CACCG was added to the 3’ end of the forward sgRNA sequence and AAAC and C were added to the 5’ and 3’ ends, respectively of the reverse sgRNA sequence.

### Cloning of sgRNA-specific oligonucleotides into pCRISPR-EASY

Complementary oligonucleotides for each sgRNA were combined at equal molar ratios (10 uM each), heated at 95˚C for 5 min and then gradually cooled to room temperature. The resulting DNA fragments were ligated into *Bsm*BI digested and gel extracted pCRISPR-EASY using T4 DNA ligase and then introduced into *E*. *coli* strain DH5a λ*pir* [34]. *E*. *coli* transformants harboring pCRISPR-EASY were screened by colony PCR using oligonucleotides sgRNAseqF and sgRNA-specific reverse oligonucleotides (S1 Table) and confirmed by sequencing.

### Introducing pCRISPR-EASY::sgRNA into RAW264.7 cells

Plasmids were transformed into RAW264.7 cells as previously described [35] with the following modifications. Briefly, RAW264.7 cells were resuspended in DMEM containing 10% FBS at a concentration of 3.75×10^7^ cells/ml. 12–24 μg of plasmid in 50 μl of 1× phosphate buffered saline (PBS) was combined with 200 μl of RAW264.7 cells (7.5x10^6^ cells) and incubated at room temperature (RT) for 5 min. Cells were electroporated at 250 volts, 950 μF capacitance and ∞ resistance, incubated at RT for 10 minutes and then combined with 5 ml of culture medium. Cells were immediately centrifuged at 234 ×*g* for 5 min at RT, resuspended in 10 ml of fresh culture medium, then transferred to a 10-cm tissue culture-treated Petri dish and incubated at 37°C for 24 hours. The medium was then replaced with 10 ml of fresh medium containing 10 μg/ml of blasticidin and incubated for 7 days, replacing the medium with fresh selective medium every 2–3 days.

### Isolation of CRISPR-Cas9 edited monoclonal cell lines by limiting dilution

To isolate individual monoclonal cell lines, blasticidin resistant isolates were pooled, serially diluted to 20 cells/ml in culture medium containing 10 μg/ml of blasticidin and then 0.1 ml samples were aliquoted into individual wells of a 96-well plate and incubated at 37°C for 12 days. For each individual sgRNA target, eight monoclonal cell lines were harvested using Trypsin-EDTA treatment and transferred to a 96-well plate containing 200 ul of culture medium and expanded for 2–3 additional days until 70–80% confluent. Cells were then harvested in 200 ul of spent medium. 50 ul of the cell suspension was combined with DMSO to a 10% final concentration and stored at -80°C and the remaining 150 ul of cell suspension was used to screen cells for gene editing (below).

### Screening of monoclonal cell lines for CRISPR-Cas9 target gene mutation

The presence of indels in the target cleavage sites were determined using the Surveyor™ Mutation Detection kit (IDT, Coralville, IA) and/or PCR amplification of target genes and Sanger sequencing. Genomic DNA was isolated from 1×10^7^ wild type RAW264.7 cells using a DNeasy Blood and Tissue kit (QIAGEN). Genomic DNA from candidate CRISPR-Cas9 knockout monoclonal cell lines was isolated by combining 150 ul of the monoclonal cell suspension harvested from a monoclone expansion (above) with 30 μl of QuickExtract DNA Extraction Solution (Lucigen) and incubating at 65°C for 10 minutes, 98°C for 2 minutes and then cooling to 4°C, as per the manufacturer’s instructions. Sites of genome editing were amplified by PCR and then analyzed for mutations. For the Surveyor endonuclease assay, equimolar amounts of cleavage site-specific PCR amplicons derived from wild type and CRISPR-Cas9 edited cells as well as homo- and hetero-duplexes of control ‘C’ and ‘G’ amplicons were hybridized, treated with Surveyor endonuclease following the manufacturer’s instructions and the presence of digested amplicons due to indels were determined by agarose gel electrophoresis. To verify genome editing at target sites, PCR amplicons were sequenced.

### Analysis of CRISPR-Cas9 genome editing

To identify CRISPR-Cas9-mediated genome modifications, the sequences of sgRNA target site PCR amplicons were analyzed using the Inference of CRISPR Edits (ICE) tool (Synthego Corporation) (https://ice.synthego.com/#/) [36]. For those candidates with indels in the target gene in both chromosomes and predicted to result in loss of the encoded protein, the corresponding monoclonal cell lines stored at -80°C (above) were expanded, target protein depletion was verified by Western analysis.

### Verification of target protein depletion by Western analysis

1×10^7^ wild type and CRISPR-Cas9 edited monoclonal cells were resuspended in 200 μl of 2× Laemmli sample buffer [100 mM Tris-Cl (pH 6.8), 4% (w/v) sodium dodecyl sulfate (SDS), 0.2% (w/v) bromophenol blue, 20% (v/v) glycerol and 200 mM dithiothreitol] and heated at 95°C for 10 minutes. 2–20 μl of cell lysate was separated by SDS-PAGE, transferred to nitrocellulose membrane and block with 5% non-fat milk in 1× PBS containing 0.05% Tween-20. Membranes were then probed with α-Tubulin antibody (1:20,000–1:500,000, Sigma) and the appropriate primary antibody (α-PMP70 (1:500, Abcam), PEX5 (1:1,000, Novus), or α-PEX19 (1:500, ThermoFisher Scientific) for 6 hrs at RT or overnight at 4°C followed by secondary antibody-conjugated horseradish peroxidase (HRP) for 1–2 hrs at RT. Proteins were detected with Amersham ECL™ Start Western Detection Reagent (GE Healthcare) and imaged using a AI600 Gel Imaging System (GE Healthcare).

### A step-by-step protocol

A detailed, step-by-step protocol from sgRNA oligonucleotide design to monoclonal cell line screening is provided in S1 File Protocol and has been deposited in protocols.io at doi.org/10.17504/protocols.io.rm7vzxzxrgx1/v1.

## Results

### A highly efficiency cloning plasmid for delivery of sgRNA and CRISPR-Cas9

**Fig 1:**
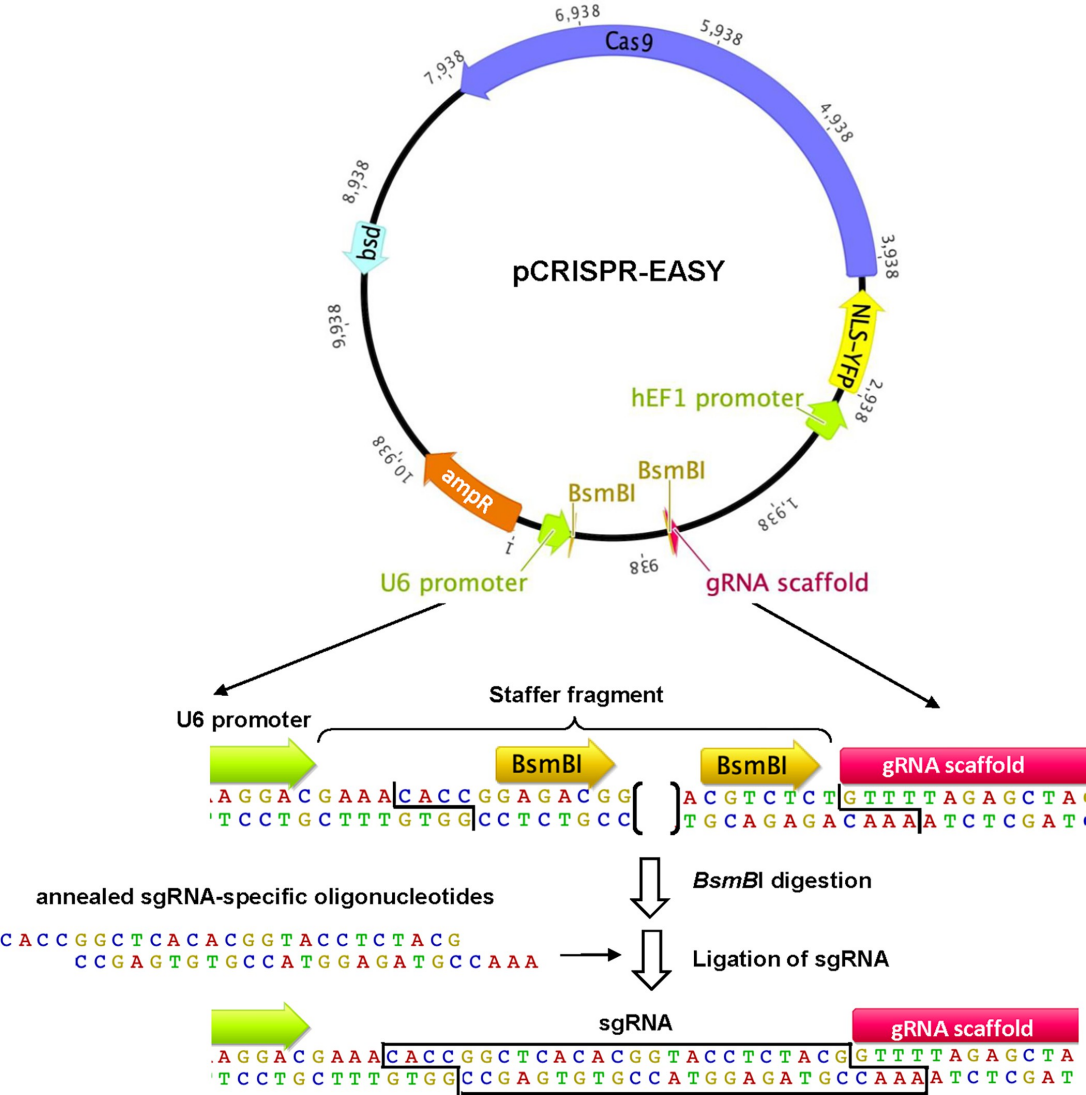
pCRISPR-EASY. pCRISPR-EASY encodes YFP-2a-Cas9 under the control of the human EF1 promoter and a guide RNA scaffold. sgRNAs are incorporated under the control of a human RNA polymerase II U6 promoter through *Bsm*BI digestion-mediate replacement of a staffer fragment with sgRNA-specific annealed oligonucleotides. The resulting plasmid pCRISPR-EASY::sgRNA is a dual sgRNA CRISPR-Cas9 delivery system. Bsd, blasticidin resistance gene.

**Fig 2:**
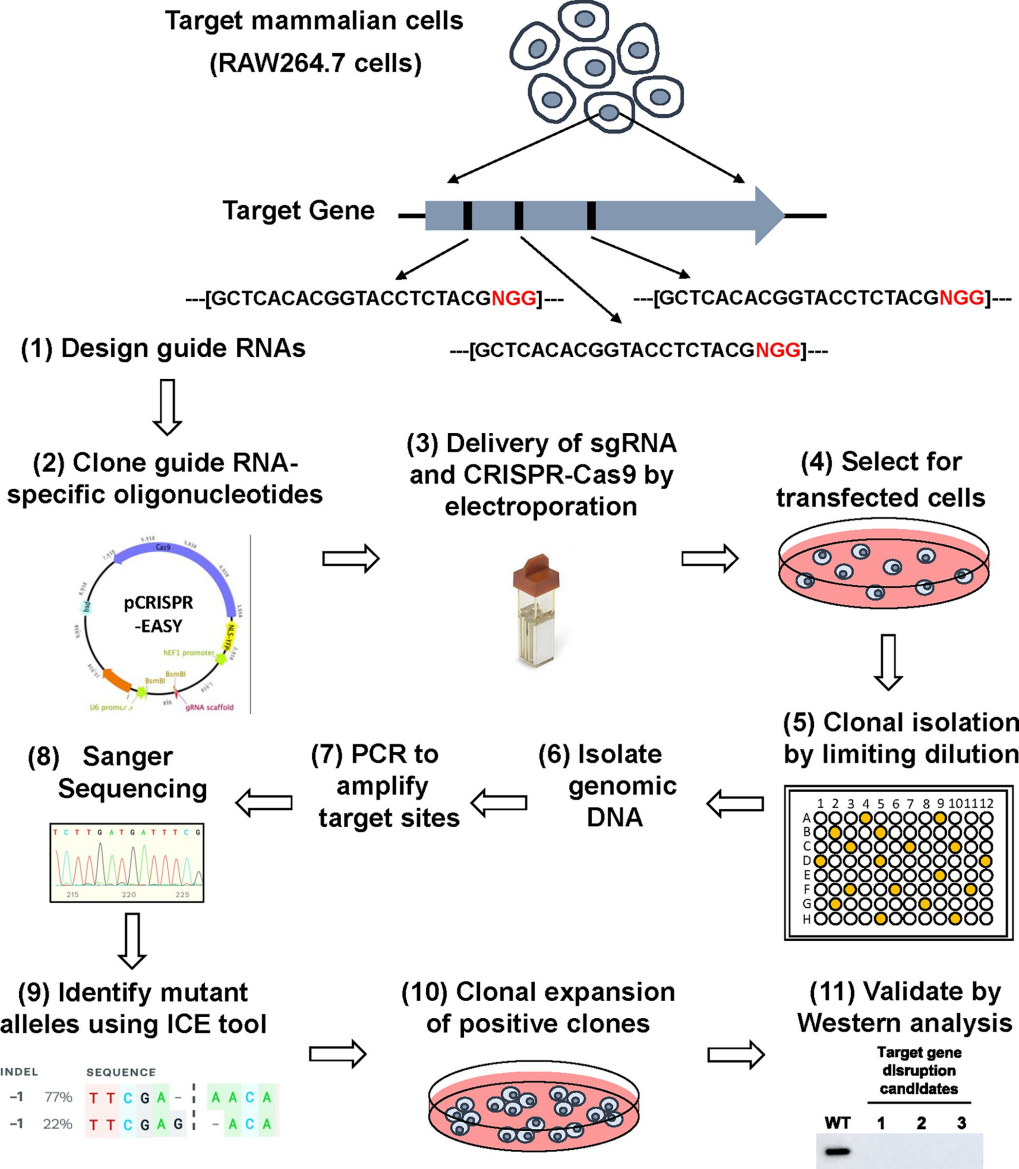
A comprehensive guide for genome editing in RAW267.4 cells using pCRISPR-EASY. 1) sgRNA-specific oligonucleotides are designed using the GPP sgRNA designer tool. 2) sgRNA oligonucleotide pairs are annealed and cloned into pCRISPR-EASY. 3) sgRNA containing plasmids are introduced into RAW267.4 cells by electroporation. 4) Transfected cells are selected for with blasticidin. 5) Individual clones are isolated and expanded limiting dilutions (or by fluorescence-activated cell sorting). 6) Genomic DNA is isolated with a single-step procedure using QuickExtract DNA Extraction Solution. 7) Target sites of genome editing are amplified by PCR. 8) PCR amplicons are Sanger sequenced and then 9) analyzed using the ICE tool [36] to identify indels. 10) Individual monoclonal cell lines with null alleles are expanded. 11) Depletion of the encoded proteins is verified by Western analysis. A step-by-step protocol is provided in S1 File Protocol.

A single plasmid harboring a sgRNA and CRISPR-Cas9 is the most efficient way to simultaneously introduce both into a target cell for genome editing. One such plasmid, pEF6-nls-YFP-2A-Cas9 [30] has been successfully used for CRISPR-Cas9-mediated genome editing. This plasmid encodes hCas9 fused to YFP which allows visualization and sorting of transfected cells using fluorescence microscopy and fluorescence-activated cell sorting (FACS). However, introducing a guide RNA scaffold into this plasmid requires *de novo* synthesis and cloning of a 450 bp gBlock that consists of a U6 promoter, target sgRNA sequence, guide RNA scaffold and transcription terminator. The synthesis of complete RNA scaffolds is costly, especially if multiple genes are to be mutated. To circumvent this, many CRIPSR-Cas9 plasmids are designed to allow a 20 bp target oligonucleotides to be cloned into a sgRNA scaffold already present within the plasmid [37]. However, these are often plagued by poor cloning efficiency. To address these issues, a DNA fragment was introduced into pEF6-nls-YFP-2A-Cas9 that consists of a U6 promoter, guide RNA scaffold, and transcription terminator that are separated by a staffer fragment [32] to generate pCRISPR-EASY (Fig 1). The staffer fragment is flanked by *Bsm*BI restriction sites allowing for efficient replacement with a small oligonucleotide sgRNA sequence (Fig 1). Based on previous reports that shorter (25 bp) staffer fragments result in low efficiency of sgRNA cloning [38], a larger (745 bp) staffer fragment was used [39]. To test the efficiency of cloning, the introduction of 3 sgRNAs for each of 7 target genes (*Pmp70*, *Pex5*, *Pex7*, *Pex19*, *Gnpat* and *Dhrs7b/Pexrap*) (Table 1) into pCRISPR-EASY (Fig 2, Steps 1–2) was examined. For each of the 21 sgRNAs, we screened four individual isolates and found that for 20/21 sgRNAs all four isolates (100%) contained an insert and for 1 sgRNA, 3/4 isolates (75%) contained an insert (data not shown). A single isolate for each sgRNA was verified by sequencing. These findings demonstrated that pCRISPR-EASY allows for efficient, cost-effective generation of a dual sgRNA CRISPR-Cas9 delivery plasmid.

**Table 1:** Efficiency of genome editing using pCRISPR-EASY in RAW264.7 cells.

Targeted Gene	sgRNA	No. clones sequenced	Clones with indels (nature of the indel)^a^	Clones without indels	% Clones with indels
*Pmp70*	sgRNA-1	3	A1 (-2, -8) D1 (+1) H1 (-2, -8)		100
	sgRNA-2	2	A1 (-2, -28, -4, -3, -1, -26) E1 (0, -2, -1, +1)		100
*Pex5*	sgRNA-1	4	A1 (+12, -4, +2, +1, -10, -8, -6, -3, -1, -30, -21, -18, -10, -14, -9) C1 (+12, -4, +1, -10, -8, -6, -3, -1, +2, -30, -21, -18, -12, -14, -9) D1 (-1)	B1	75
	sgRNA-2	3	B3 (+15, -16, -23) C3 (-4, -2, -4)	E3	75
	sgRNA-3	4	C5 (-17, -17, -17) E5 (-17)	B5, D5	50
*Gnpat*	sgRNA-1	2	C7 (-8) D7 (-8)		100
	sgRNA-2	3		B9, C9, D9	0
	sgRNA-3	4	F11 (+8, +5, -18, -24, -5, -4, 0)	A11, B11, E11	25
*Pex19*	sgRNA-1	4	B1 (+1) D1 (-10, -10, -10, -10)	C1, E1	50
	sgRNA-2	4	B6 (-11, -1, -1, -12, -12) C6 (-1, -1) D6 (-2) E6 (-1, -2, -2, -1)		100
	sgRNA-3	4	A12 (-2) C12 (0, -6, -6, -6) D12 (-3)	B12	75
*Pexrap*	sgRNA-1	4	A1 (-4, -4) B1 (0, +1, -1) C1 (+4, -15) D1 (-2)		100
	sgRNA-2	4	F6 (-1)	A6, B6, E6	25
	sgRNA-3	4	B12 (-2, 0)	C12, D12, E12	25
*Pex7*	sgRNA-1	4		B1, C1, D1, E1	0
	sgRNA-2	4	C6 (-1) G6 (+1)	E6, F6	50
	sgRNA-3	4	B12 (-1)	C12, D12, E12	25
*Pex10*	sgRNA-1	4	B1 (+1, -4, -4, -4) C1 (0, -1, -1)	A1, D1	50
	sgRNA-2	4	A6 (-1, -4, -4, -4, -2) E6 (+1, -1) F6 (-5) H6 (-5, +1)		100
	sgRNA-3	4		A12, B12, C12, E12	0

^a^The nature of the indels are noted in parentheses with—and + indicating nucleotide deletion and insertions, respectively.

### Delivery of pCRISPR-EASY into macrophages by electroporation

The implementation of CRISPR-Cas9-mediated genome editing in macrophages depends on a robust sgRNA and CRISPR-Cas9 delivery system. RAW264.7 cells [25] are model mouse macrophage cell line used in research [26]. Several viral transduction and commercial transfecting agents have been used to introduce DNA into RAW264.7 cells. However, viral delivery increases the frequency of additional undesirable mutations [40] and is time-consuming while commercially available transfecting reagents are not cost-effective, particularly when many genes are targeted for mutation. To establish a highly efficient and cost-effective genome editing protocol, we have defined conditions for electroporation of pCRISPR-EASY into RAW264.7 cells, circumventing the need for viral packaging or transfection reagent. To demonstrate the efficiency of this approach, we individually electroporated each of the 21 sgRNA-harboring plasmids into RAW264.7 macrophages, and transfected cells were selected for with blasticidin (Fig 2, Steps 3–4). Antibiotic resistant isolates were obtained for all 21 sgRNAs. We then screened 16 individual blasticidin resistant monoclones generated using *Pmp70* sgRNA-1 by PCR and determined that 14 (87.5%) had the sgRNA harboring plasmid integrated into the genome (S1 Fig). These results demonstrated that electroporation of pCRISPR-EASY-based plasmids into RAW264.7 macrophages is an efficient method for their stable incorporation into these cells.

### Isolation of CRISPR-Cas9 edited monoclonal cell lines by limiting dilutions

Although heterogeneous populations of knockout cells are advantageous in many downstream applications, the systematic investigation of individual genes requires the isolation of monoclonal cell lines. Fluorescent assisted cell sorting (FACS), cloning cylinder, agarose cloning and colony picking robots [41–45] are commonly used techniques to isolate individual cells for clonal expansion. Indeed, pCRISPR-EASY is designed to allow cells harboring the plasmid to be isolated by FACS based on the fluorescence of the YPF:Cas9 fusion protein. However, these methods require specialized, often expensive equipment and expertise. As an alternative, we outline guidelines and test the efficacy of using limiting dilution to isolate monoclonal cell lines (Fig 2, Step 5) as a more cost-effective approach. To do this, blasticidin resistant RAW264.7 cells for each of the 21 sgRNAs were harvested, diluted and aliquoted in a 96-well plate. After clonal expansion in selective medium, an average of 25 (ranging from 17 to 34) monoclonal cells per 96 well plate (26%) grew out. Thus, the high efficiency of transfection with pCRISPR-EASY enables the use of limiting dilution to isolate large numbers of monoclonal cell lines.

### Identifying CRISPR-Cas9 genome modifications in RAW264.7 cells

**Fig 3:**
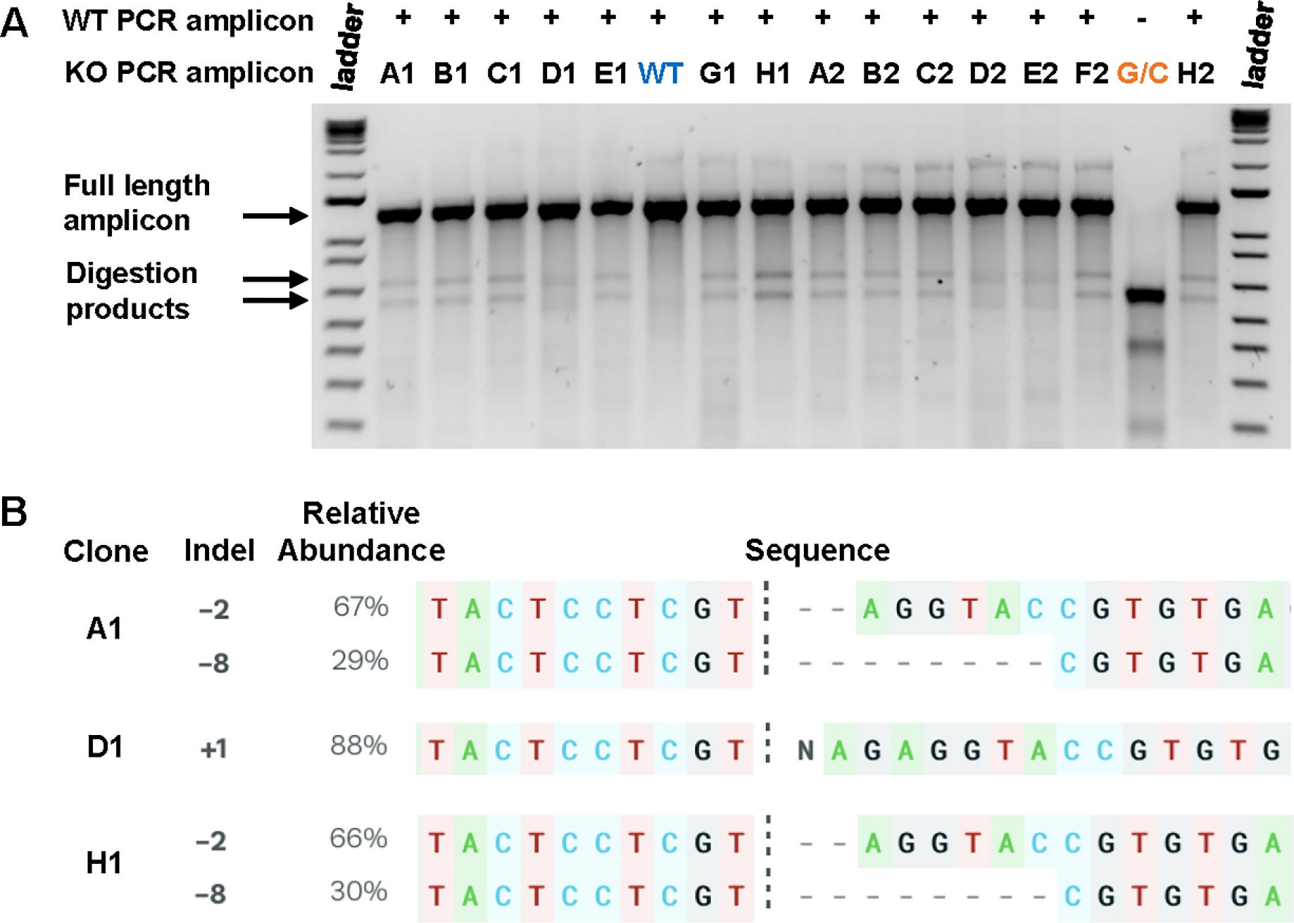
Characterization of indels in monoclonal cell lines. **A)** Surveyor endonuclease assay demonstrates the presence and efficiency of generating indels in RAW264.7 cells. Fourteen individual monoclones generated using *Pmp70* sgRNA-1 (S1 Table) were analyzed. Cleavage of wild type (WT)—mutant hybrid PCR amplicons indicates the presence of indels. Individual monoclones are designated as alpha-numeric symbols corresponding to their position in the 96-well plate. G/C, positive control PCR products G and C provided by Surveyor™ Mutation Detection kit. KO, knockout. **B)** Identification of monoclones with mutations in *Pmp70* using the inference of CRISPR edits (ICE) tool [36] demonstrates the presence of both insertions and deletions at the target site that result in frame shift mutations coding for premature translational termination.

Genome editing using CRISPR-Cas9 can result in a diverse array of genetic modifications at the target site [46], which may but do not always result in loss of protein production and thus can greatly impact the interpretation of downstream experiments. One feature that distinguishes pCRISPR-EASY from other commonly used plasmids such as pX330 [7], is that in pCRISPR-EASY, Cas9 expression is under the control of the human elongation factor-1alpha (hEF1a) promoter which has been shown to be more active than other commonly used promoters, such as the CMV enhancer/Chicken beta-actin and rabbit beta-globin splice acceptor site (CAG) promoter [47]. To determine the efficiency of genome editing using pCRISPR-EASY, we targeted 7 different genes and then characterized the nature of indels in our isolated monoclonal cell lines. First, we examined monoclonal cell lines generated for a single gene, *Pmp70* using two sgRNAs (sgRNA-1 and sgRNA-2) (Table 1) with the Surveyor endonuclease assay [48]. To facilitate the high throughput screening of large numbers of monoclonal cell lines, we streamlined the template preparation procedure by adapting a single-step genomic DNA extraction technique that uses QuickExtract DNA extraction solution [49] (Fig 2, Step 6). Analyzing the PCR amplicons with Surveyor endonuclease for 14 and 6 monoclonal cell lines generated using sgRNA-1 and sgRNA-2, respectively, demonstrated that 19/20 (95%) contained genome modifications in *Pmp70* (Fig 3A and data not shown). Sequencing of the targeted region and analysis using the ICE tool [36] (Fig 2, Steps 7–9) for 5 of the monoclonal cell lines demonstrated that all 5 code for indels at the target site that result in frame shifts and premature truncation of the encoded protein (Table 1 and Fig 3B).

**Fig 4:**
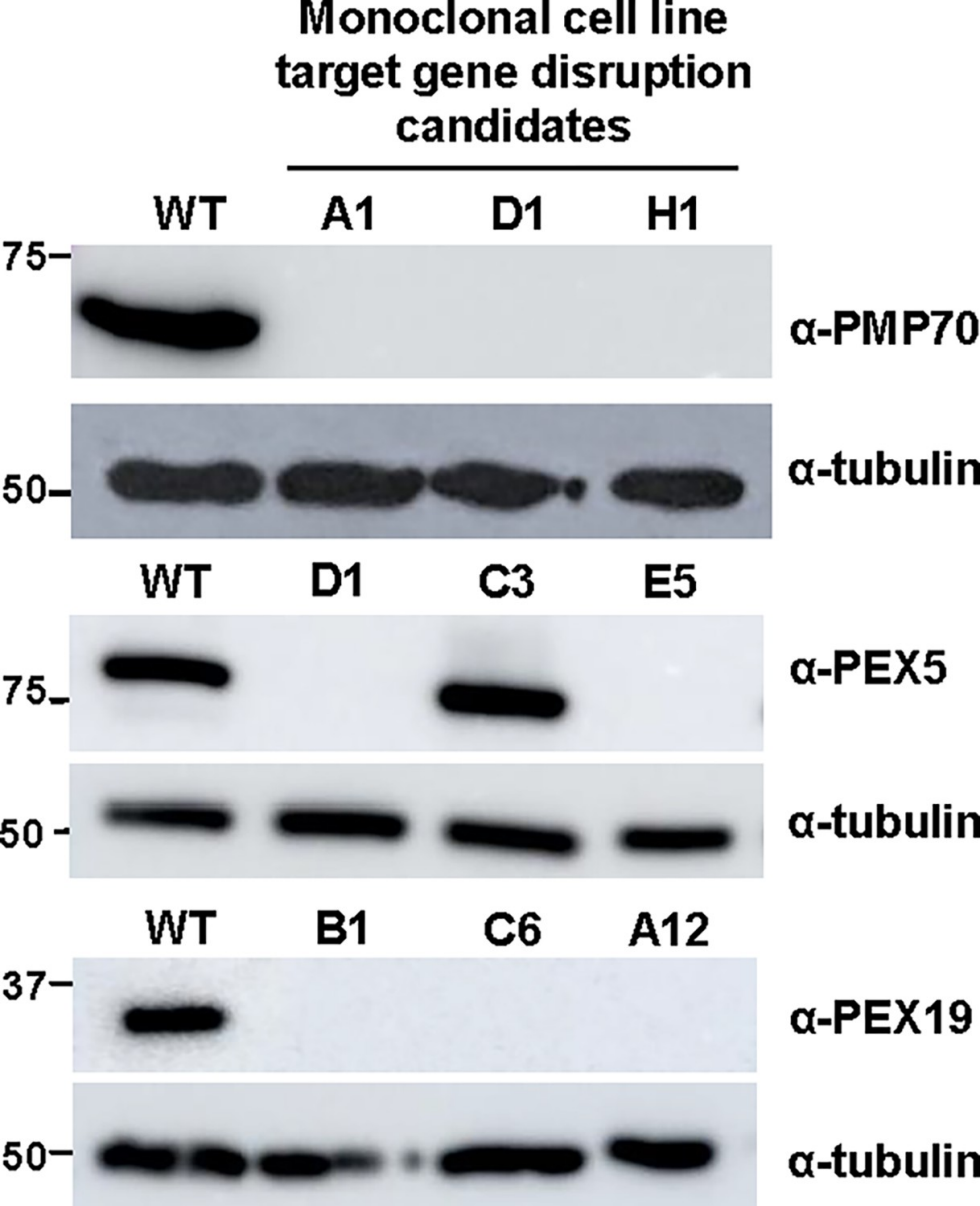
Verification of target gene null alleles in RAW264.7 monoclonal cell lines. Western analysis of whole cell lysates of wild type RAW264.7 macrophages and candidate CRISPR-Cas9 edited monoclonal cell lines demonstrate the depletion of target gene encoded proteins. Tubulin was used as a loading control.

The high frequency of monoclonal cell lines with indels in *Pmp70* demonstrated the high efficiency of genome editing using pCRISPR-EASY-based plasmids and an opportunity to develop a more high-throughput screening protocol that circumvents the need for enzyme mismatch cleavage-based assays for detecting indels [48,50] while still defining the precise nature of the mutations generated. To do this, universal oligonucleotide design parameters and PCR amplification conditions were optimized to consistently generate a single PCR amplicon of sgRNA target sites for all sgRNAs (S1 File Protocol). This allows PCR amplicons to be Sanger sequenced directly [51] bypassing the need for gel purification or cloning into a plasmid prior to Sanger [52,53] or next-generation [54] sequencing. Using this method, we analyzed indels of 2–4 individual monoclonal cell lines for the remaining 20 sgRNAs encompassing the 7 genes targeted (Fig 2, Steps 7–9). Of the total 21 sgRNAs used for genome editing, 17 (81%) yielded indels in their target genes (Table 1) with at least 2 of the 3 sgRNA for each gene resulted in genome editing. Of the 73 monoclonal cell lines analyzed by sequencing (2–4 monoclones per sgRNA), 39 (53%) contained mutations with a minimum of two different mutations obtained for each gene targeted (Table 1). Importantly, 68% of the indels were predicted to disrupt the target gene and thus, cause depletion of the encoded protein (Table 1). To verify this, protein production for 3 individual monoclonal cells lines for *Pmp70*, *Pex5* and *Pex19* target genes was determine by Western analysis (Fig 2, Step 11). Of the 9 clones tested, 8 (~90%) showed robust depletion of the target protein when compared to wild type RAW264.7 cells (Figs 4 and S2). Collectively, these results define a robust, cost-effective method for CRISPR-Cas9-mediated genome editing in RAW264.7 cells.

## Discussion

CRISPR has revolutionized genome editing however, like many technological advances, the utility and practical application can vary between systems. Here we present a simple, highly efficient and economically feasible protocol for genome editing in RAW264.7 cells. The newly engineered vector pCRISPR-EASY allows for nearly 100% efficient and cost-effective cloning of 20 bp sgRNA and simultaneous introduction of sgRNA and CRISPR-Cas9 into target cells. Using a simple electroporation procedure, we demonstrate efficient incorporation of this plasmid into RAW264.7 cells that circumvents the need for lentiviral packaging or commercial transfection reagents. Although the pCRISPR-EASY enables the use of fluorescence as a reporter to screen and sort stably transfected cell expressing YFP:Cas9, the high frequency of transfection and genome editing renders limiting dilution highly feasible for isolating desired clones. To further facilitate the utility of this system, we have established a simplified procedure for screening indels in candidate monoclonal cell lines, including a streamlined genomic DNA extraction protocol for large numbers of candidates and PCR target site amplification parameters that enable direct sequencing in the absence of further purification or cloning of PCR amplicons [52,53]. Furthermore, the efficiency of this system makes PCR amplicon sequencing and analysis with the ICE tool [36] sufficient for characterizing CRISPR-Cas9 generated indels. Although this procedure was designed for genome editing in RAW264.7 cells, it can easily be adapted for use in other cell types.

## Conclusions

We present an economical, highly efficient, reproducible and facile protocol for genome modification in RAW264.7 cells using CRISPR-Cas9, including a streamlined, step-by-step protocol. The protocol takes approximately 5–6 weeks from start to finish allowing for targeted disruption of multiple genes simultaneously in a short period of time without the burden of costly reagents or specialized equipment. We describe the application of this method for genome editing in RAW264.7 macrophages, which can easily be adapted for use in other mammalian cells.

## Supporting information

S1 Fig Characterization of stable incorporation of pCRISPR-EASY into the genome in blasticidin resistant monoclonal cell lines. PCR amplification of a region of pCRISPR-EASY from isolated genomic DNA of was used to assay for chromosomal integration of pCRISPR-EASY in 14 individual blasticidin resistant monoclonal RAW264.7 cell lines generated using *Pmp70* sgRNA-1 (S1 Table). pCRISPR-EASY was used as a positive control.
(TIF)

S2 Fig Verification of target gene null alleles in monoclonal cell lines. Full images of Western analysis shown in Fig 4 examining the depletion of target gene encoded proteins in candidate CRISPR-Cas9 edited RAW264.7 monoclonal cell lines when compared to wild type cells. Tubulin was used as a loading control.
(TIF)

S1 File Protocol A step-by-step protocol for genome editing in RAW264.7 cells using pCRISPR-EASY. (PDF)

S1 Table Oligonucleotides. (PDF)

S2 Table Plasmids. (PDF)
