# Supplementary figures and images for: An efficient and cost-effective method for disrupting genes in RAW264.7 macrophages using CRISPR-Cas9
Source: PLoS One. 2024 Mar 14;19(3):e0299513. doi: 10.1371/journal.pone.0299513 (PMC10939251; doi:10.1371/journal.pone.0299513)

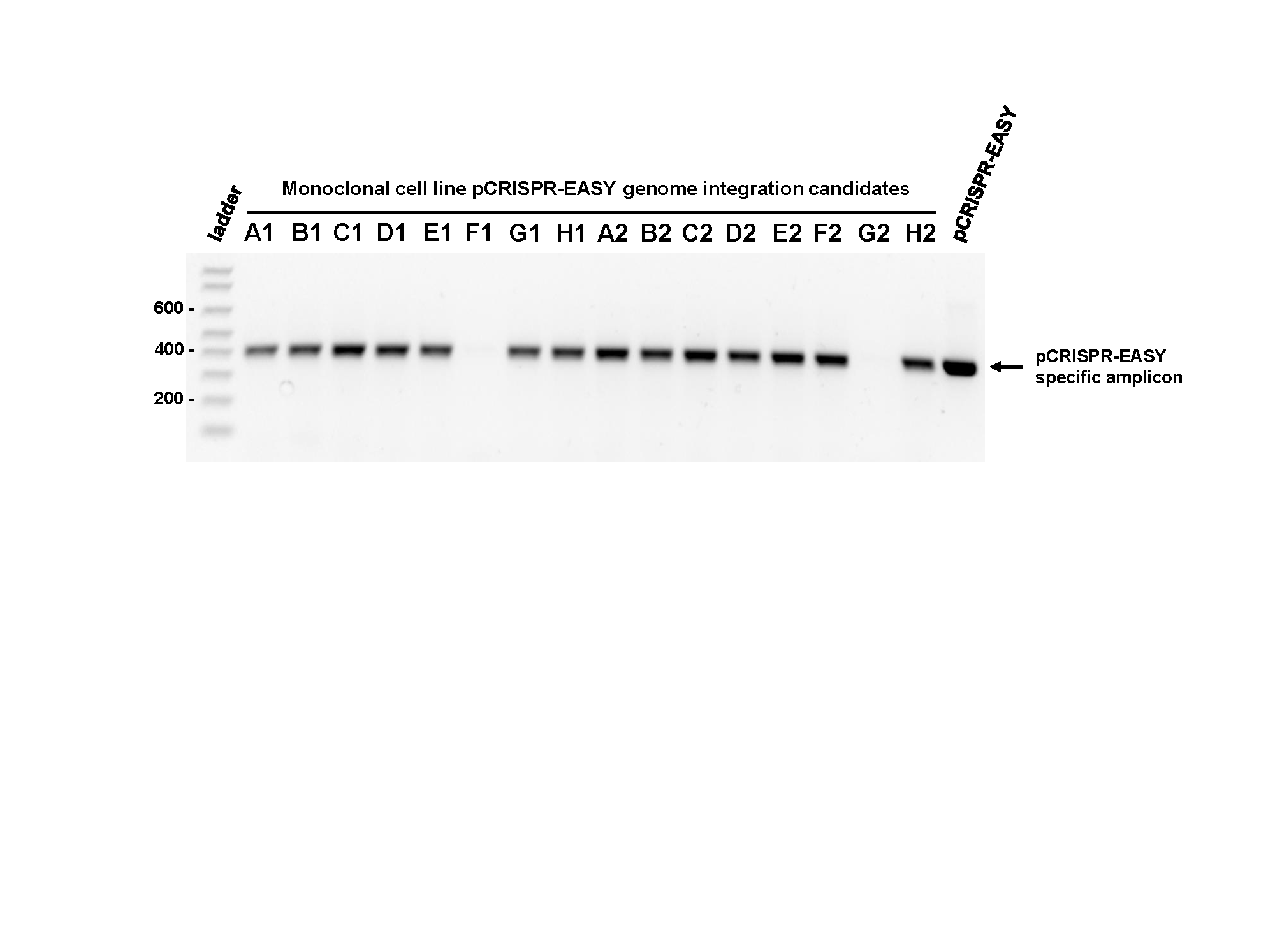

Supplement: S1 Fig — PCR amplification of a region of pCRISPR-EASY from isolated genomic DNA of was used to assay for chromosomal integration of pCRISPR-EASY in 14 individual blasticidin resistant monoclonal RAW264.7 cell lines generated using Pmp70 sgRNA-1 (S1 Table). pCRISPR-EASY was used as a positive control. (TIF) [file pone.0299513.s001.tif]

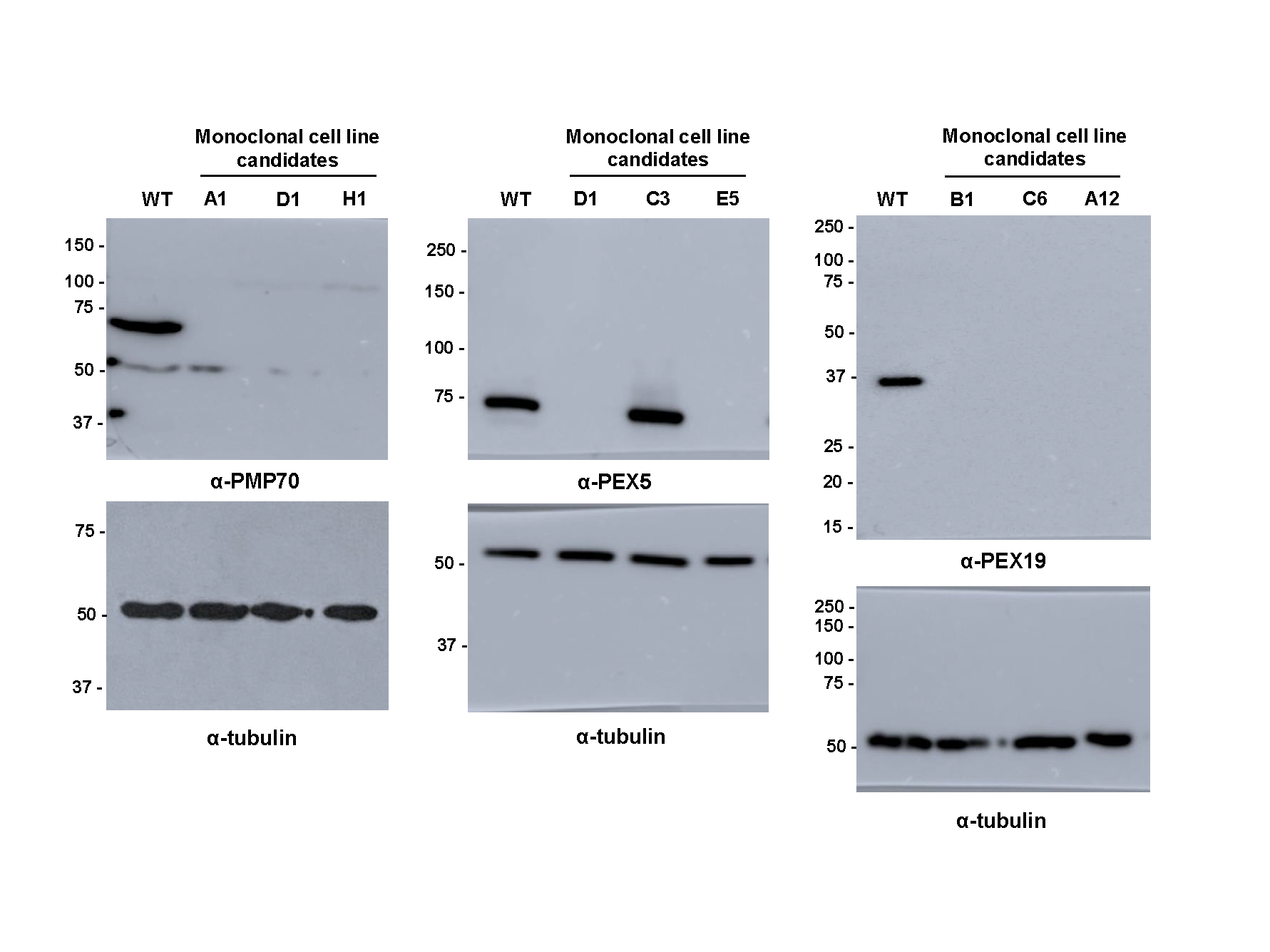

Supplement: S2 Fig — Full images of Western analysis shown in Fig 4 examining the depletion of target gene encoded proteins in candidate CRISPR-Cas9 edited RAW264.7 monoclonal cell lines when compared to wild type cells. Tubulin was used as a loading control. (TIF) [file pone.0299513.s002.tif]
