# Supplementary material for: An efficient and cost-effective method for disrupting genes in RAW264.7 macrophages using CRISPR-Cas9
Source: PLoS One. 2024 Mar 14;19(3):e0299513. doi: 10.1371/journal.pone.0299513 (PMC10939251; doi:10.1371/journal.pone.0299513)
Supplement: S1 File Protocol — (PDF) [file pone.0299513.s003.pdf]

S1 File Protocol

Step-by-step protocol for genome editing in RAW264.7 cells using pCRISPR-EASY

A. Design guide RNAs

1) Predict sgRNA(s) using the GPP Web Portal: https://portals.broadinstitute.org/gpp/public/analysis-tools/sgrna-design
2) Under the 'CRISPRko' tab,
   a) In the "CRISPR Enzyme" drop-down menu, select 'SpyoCas9 (NGG)'.
   b) In the "Target Genome" drop-down menu, select 'Mouse GRCm38 (NCBI RefSeq v.108)' for RAW264.7 cells. The lack of complete genome sequence for the RAW264.7 cell line can be compensated for by using the genome sequence of C57BL/6 mice.
3) In the "Specify Target(s)" box, enter the Gene ID(s) to be targeted.
   Gene ID(s) can be found through GenBank: https://www.ncbi.nlm.nih.gov/genbank/
   a) Select 'Gene' from the drop-down menu
   b) Enter the gene name and source species. For example, PMP70 mouse.
   c) Hit 'Search' and collect the GeneID number from the display box. For example, 19299.
4) In the 'reCAPACHA' box, click 'I am not a robot' and select 'Submit' for results.
5) Download the 'sgRNA Picking Results': this will provide a tab-delimited text file of candidate sgRNAs.
6) Select three target sgRNA sequences from the column designated 'sgRNA sequence': these are the gene specific Forward oligonucleotides (20 nucleotides in length).
   Note: To maximize the probability of genome editing, select a minimum of three sgRNA sequences, beginning with the top picks in the column labeled 'Pick Order' (sgRNAs 1, 2 and 3). Typically, at least 2/3 sgRNAs result in genome editing.
7) Generate the complementary Reverse oligonucleotide using your preferred DNA sequence editing tool. For example: https://www.bioinformatics.org/sms/rev_comp.html.
8) For each set of homologous sgRNA Forward and Reverse oligonucleotide pairs, add the cohesive ends of *BsmB*I restriction sequence to facilitate cloning:
   a) For the Forward oligonucleotide, add '*caccg'* to the 5' end of the oligonucleotide sequence. For example, for sgRNA sequence 5'-GCTCACACGGTACCTCTACG-3', the Forward oligonucleotide will be 5'-*caccg*GCTCACACGGTACCTCTACG-3'.
   b) For the Reverse oligonucleotide, add '*aaac'* to the 5' end and '*c*' to the 3′ end. For example, sgRNA sequence 5'-CGTAGAGGTACCGTGTGAGC-3', the Reverse oligonucleotide will be 5'-*aaac*CGTAGAGGTACCGTGTGAGCc-3'.
9) Order oligonucleotides from your supplier of choice.
10) Resuspend the oligonucleotides in nuclease free dH_2O at a final concentration of 100 µM.

B. Cloning of guide RNA specific oligonucleotides into plasmid pCRISPR-EASY

pCRISPR-EASY can be isolated from *Escherichia coli* strain TO1463 using standard plasmid isolation procedure or kit such as the QIAprep Spin Miniprep kit (QIAGEN). It is recommended to elute the plasmid with nuclease free H_2O.

1) Digest 5 µg of pCRISPR-EASY with *Bsm*BI as follows:

pCRISPR-EASY	x µl
10× NEB buffer 3.1	5 µl
*BsmB*I (NEB)	1 µl
Nuclease free dH_2O	44-x µl
Total	50 µl

2) Incubate the digestion reaction at 55°C for 1 hour in a water bath or Thermocycler.

Note: Digestion of pCRISPR-EASY with *Bsm*BI yields two DNA fragments: 11,192 bp (the plasmid backbone encoding YFP-Cas9 and sgRNA scaffold) and 745 bp (the staffer fragment).

3) Separate the digestion reaction in a 0.7% agarose gel and gel purify the larger 11,192 bp fragment using for example, the Wizard® SV PCR and Gel Clean-up System (Promega).
4) Anneal the Forward and Reversed oligonucleotides (Section A, Step 10) by combining equimolar amounts in a PCR tube (final concentration of 10 µM each) and incubating in a Thermocycler at 95°C for 5 min, then cool to 25°C at 0.1°C/sec (or turn off the Thermocycler and allow it to cool gradually to room temperature):

10× T4 DNA Ligase buffer	1 µl
100 µM Forward oligonucleotide	1 µl
100 µM Reverse oligonucleotide	1 µl
Nuclease free H_2O	7 µl
Total	10 µl

5) Dilute the annealed oligonucleotides 1:200 by adding 1 µl of annealed oligonucleotides to 199 µl nuclease free H_2O.
6) Set up a ligation reaction by combining the following components in an Eppendorf tube and incubate at 25°C for 1 hour. 10 ng of *Bsm*BI digested pCRISPR-EASY will provide hundreds of colonies with cloned inserts.

*Bsm*BI digested pCRISPR-EASY	x µl
10× T4 DNA Ligase buffer (NEB)	1 µl
T4 DNA Ligase (NEB)	1 µl
Annealed diluted oligonucleotides	1 µl
Nuclease free H_2O	7-x µl
Total	10 µl

7) Heat inactivate the ligase by incubating at 65°C for 10 min then cool the reaction on ice.
8) Transform 5 µl of the ligation reaction into *E. coli*.

   Note: For these studies, we used either *E. coli* HST08 Stellar™ Competent Cells (Takara Bio) or *E. coli* DH5α λ*pir* chemically ultracompetent cells prepared by the Inoue method (https://cshlpress.com/pdf/CondProt.pdf), heat shocking for 45 seconds at 42°C and recovering in 1 ml of SOC medium at 37°C for 1 hour. Plating 100 µl (1/10 of the transformation) on solid LB medium supplemented with 50 µg/ml of carbenicillin typically yields approximately 50 colonies. We expect that using any other strain of highly competent *E. coli* would provide similar outcomes.

9) Screen *E. coli* transformants for sgRNA containing clones by colony PCR as follows:
   a) Set up the following PCR reaction per four *E. coli* transformants to be screened:

2× EconoTaq® Plus Green Master Mix (Lucigen)	55.0 µl
Nuclease free H_2O	54.4 µl
100 µM sgRNAseqF	0.3 µl
100 µM Reversed oligonucleotide specific to sgRNA	0.3 µl
Total	110.0 µl

   b) Split the PCR reaction equally into four individual PCR tubes, each with 25 µl.
   c) For each transformant to be screened, add ½ of a colony to a single PCR tube.

d) Amplify the sgRNA insertion site in a Thermocycler using the following parameters:
   94°C, 5 min
   94°C, 30 sec; 50°C, 30 sec; 72°C, 30 sec x 35 cycles
   72°C, 2 min
   12°C, ∞
e) Separate 10 µl of each PCR reaction in a 2% agarose gel supplemented with SYBR Safe DNA Gel Stain (1:10,000) at 100 volts for 1 hour. Visualize on a UV box to identify positive clones - clones containing the sgRNA oligonucleotide will yield a DNA fragment of 413 bp.

10) For each sgRNA, select a positive clone and inoculate 3 ml of LB broth supplemented with 100 µg/ml of ampicillin with the remaining ½ colony (Step 9c) and incubate overnight (~16 hours) at 37°C with shaking.
11) Prepare a glycerol stock of each strain in Step 10 by combining 600 µl of culture with 400 µl of sterile 50% glycerol (final stock is in 20% glycerol) and store at -80°C.
12) From the remaining culture, isolate plasmid from 1.5 ml using standard endotoxin free plasmid isolation procedure or kit such as the PureYield Plasmid Miniprep System (Promega).
    Note: 1.5 ml of cultured *E. coli* harboring plasmid pCRISPR-EASY typically yields ~300 ng/µl of purified plasmid when eluted with 50 µl of nuclease free dH_2O and thus enough plasmid for sequencing and transfection.
13) Verify the sgRNA insert by sequencing using sgRNAseqF primer.

C. Transfect RAW264.7 cells with pCRISPR-EASY

The protocol for transfecting RAW264.7 cells (ATCC TIB-71) with pCRISPR-EASY-based plasmids is a modified version of a protocol described previously [1]. It is recommended to use RAW264.7 cells freshly thawed from storage at low passage to limit the accumulation of background mutations.

1) Culture RAW264.7 cells in DMEM containing 10% FBS to 50-70% confluency.
2) Re-suspend cells in spent medium using a cell scraper, then harvest and transfer the cell suspension to a 15 ml conical tube.
3) Centrifuge at 234 ×*g* for 5 min at room temperature (RT), aspirate the medium and re-suspend the cells in 10 ml of fresh, pre-warmed medium to homogeneity.
4) Quantify the cell using a hemocytometer and dilute the cells to 3.75×10^7/ml with DMEM + 10% FBS.
5) Transfect RAW264.7 cells with pCRISPR-EASY-based plasmid as follows:
   a) Dilute 5-20 µg of endotoxin free plasmid in 50 µl of sterile 1×PBS.
   b) Combine 50 µl of diluted plasmid with 200 µl of 3.75×10^7/ml RAW264.7 cells in a 0.4 cm cuvette and incubate at RT for 5 minutes.
   c) Electroporate the cells at 250V, 950 µF, ∞ resistance
      We use a GenePulser Xcell Electroporation System (Biorad) equipped with a CE module using the exponential decay protocol.
   d) Incubate the cell for 10 min at RT.
   e) Transfer the cells to 5 ml of DMEM + 10% FBS in a 15 ml conical tube.
   f) Centrifuge at 234 ×*g* for 5 min at RT, aspirate the supernatant and re-suspend the cell pellet with 10 ml of DMEM + 10% FBS.
   g) Transfer the cells to a 10 cm tissue culture treated Petri dish (or T175 flask) and incubate at 37°C for 24 hours.
   h) The next day, replace the medium with 10 ml of fresh, pre-warmed DMEM + 10% FBS supplemented with 10 µg/ml of blasticidin and incubate at 37°C.

i) Incubate the cells for 7 days, or until cells just begin to grow, replacing the medium with 10 ml of fresh, pre-warmed DMEM + 10% FBS + 10 µg/ml of blasticidin every 2-3 days. Note: Cells can be check for YFP:Cas9 expression using a fluorescence microscope.

6) Harvest the cells by scraping into a 15 ml conical tube, centrifuge at 234 ×*g* for 5 min at RT, aspirate the medium and re-suspend the cells in 1.5 ml of DMEM + 10% FBS to homogeneity.
7) Store cells by combining 500 µl of cell suspension with 500 µl of DMEM + 10% FBS containing 10% DMSO and freezing overnight at -80°C. The following day, transfer the tubes to the liquid nitrogen for long term storage.
8) Use the remainder of the cell suspension to isolate monoclonal cell lines in Section D below.

D. Isolation of CRISPR-Cas9 knockout monoclonal cell lines by limiting dilution.

1) Serial dilute stably transfected RAW264.7 cells (Section C) to 20 cells/ml in 12.5 ml DMEM + 10% FBS supplemented with 10 µg/ml of blasticidin.
2) Plate 100 µl aliquots into each well of a 96-well tissue culture-treated plate and incubate at 37°C for 9-12 days.
   Note: In one well of each plate, we add 100 µl of 1×10^4/ml cells to use as a reference to focus the microscope for locating monoclonal cells in the other wells (Step 3).
   Note: Alternatively, cells can be sorted and plated using fluorescence-activated cells sorting into a 96 well plate based on YFP:Cas9 expression.
3) After 3-5 days of growth, check each individual well on a daily basis for cell expansion, thoroughly monitoring the entire well to ensure outgrowth of only a single cell.
   Note: We typically use a 4× objective lens to survey the entire well for cell expansion and then thoroughly check each individual well with a 10× objective lens to ensure that wells with expanding cells only contain a single colony.
4) After 9-12 days, select 8 monoclonal cell lines for each sgRNA for further expansion and screening.
   Note: We typically screen 8 monoclonal cell lines to ensure isolation of at least one that encodes indels in both chromosomes and results in loss of protein production.
5) Aspirate the medium, add 50 µl of 0.05% trypsin-EDTA and incubate at 37°C for 20 min or until the cells begin to lift off the plate.
6) Neutralize the trypsin by adding 150 µl of DMEM + 10% FBS.
7) Re-suspend the cells by pipetting and transfer them to a new 96-well plate (200 µl total).
8) Transfer 150 µl of the cell suspension to a PCR tube and centrifuge at 234 ×*g* for 5 min at RT.
9) Aspirate the medium and resuspend the cells in 30 µl of QuickExtract™ DNA Extraction Solution (Lucigen) by pipetting several times then lyse the cells by incubating at 65°C for 10 min, 98°C for 2 min and then cooling to 4°C in a Thermocycler: this is used as template for genotyping in Section E, Step 4 below.
   Note: Cell pellets can be stored at -20°C until ready to extract DNA and genotype.
10) For the remaining 50 µl of cells in the 96-well plate (Steps 7-8), add 150 µl of fresh, pre-warmed DMEM + 10% FBS and incubate at 37°C until they reach 70-80% confluency (typically 2-3 days).
   Note: Once cells are confluent, they are expanded for long term storage (Section F) and validation of targeted gene null mutations (Section G). It typically takes 2-3 days to genotype individual monoclonal cell lines (Section E). If additional time is required, cells can be stored short term by combining the remaining 50 µl of cell suspension (Steps 7-8) with 50 µl of DMEM + 10% FBS + 20% DMSO, sealed with sterile Cold Storage Foil (USA Scientific) and stored at -80°C.

E. <u>Screening of individual monoclonal cell lines for target gene mutations.</u>

1) Extract genomic DNA (gDNA) from $1×10^7$ wild type RAW264.7 cells to use as a template for PCR amplification of sgRNA targets sites. We typically use a DNeasy Blood and Tissue kit (QIAGEN).
2) Design primers for PCR amplification of regions flanking sgRNA target sites:
   a) Download the nucleotide sequence of the mouse target gene(s) from GenBank using the gene ID used for designing sgRNA oligonucleotides (Section A, Step 3).
   b) Identify the sgRNA specific sequence within the target gene and design primers to amplify an 850-1000 bp fragment surrounding the sgRNA sequence (~400-500 bp on either side).
      For example, we use Primer3 (http://bioinfo.ut.ee/primer3-0.4.0/).
      i) Copy ~1,000 bp of sequence flanking the sgRNA into the sequence box.
      ii) Select 'Pick left primer' and 'Pick right primer' to obtain forward and reverse primers, respectively.
      iii) Set the 'Product Size Ranges' to 851-1000 bp
      iv) Set the 'General Primer Picking Conditions' to:
         Primer Size Minimum: 22, Optimum: 25, Maximum: 30
         Primer Tm Minimum: 60, Optimum: 65, Maximum: 70
         Keep the rest of the parameters as default.
      v) Select the top primer pair generated, referred to as Forward and Reverse screening primers below.

   Note: The genome sequence for the RAW264.7 cell line is not complete. In some cases, primers were designed using equivalent regions from C57B/L6 genome sequence. In some cases, this would result in multiple PCR products but redesigning primers would resolve the issue (Step 3c).
3) Test the specificity of the primer pairs and PCR conditions to generate a single PCR amplicon of the target site using the gDNA isolated from wild type RAW267.4 cells (Step 1) as template.
   a) Set up following a PCR reaction:

5× Q5 Reaction Buffer (NEB)	10.0 µl
25 mM dNTPs	0.4 µl
100 µM Forward screening primer	0.3 µl
100 µM Reverse screening primer	0.3 µl
gDNA (60 ng/µl)	1.0 µl
Nuclease free H_2O	37.5 µl
<u>Q5 High Fidelity DNA polymerase (NEB)</u>	<u>0.5 µl</u>
Total	50.0 µl

   b) PCR amplify the target sequences using the following parameters:
      98°C, 2 min
      98°C, 10 sec; 68°C, 20 sec; 72°C, 30 sec x 35 cycles
      72°C, 3 min
      12°C, ∞
   c) Separate 5 µl of the PCR reaction in 2% agarose gel supplemented with SYBR Safe DNA Gel Stain (1:10,000) at 100 volts for 1 hour and visualize on a UV box.
      Note: In ~75% of cases, a single amplicon will be generated. However, if more than one DNA band is observed, design new primer pairs and repeat Step 3. In general, we have found that altering PCR amplification parameters, including using a sequential gradient of annealing temperatures does not resolve the issue.
   d) Store the remainder of the PCR reaction at -20°C.

4) PCR amplify the sgRNA target site of the 8 individual monoclonal cell lines for each sgRNA (Section D, Step 4) to define their CRISPR-Cas9 edited indels.
   a) Set up following PCR master mix:

	1x	9x
5× Q5 Reaction Buffer (NEB)	5.00 µl	45.0 µl
25 mM dNTPs	0.20 µl	1.8 µl
100 µM Forward screening primer	0.15 µl	1.4 µl
100 µM Reverse screening primer	0.15 µl	1.4 µl
Nuclease free H_2O	9.25 µl	83.3 µl
Q5 High Fidelity DNA polymerase (NEB)	0.25 µl	2.3 µl
Total	15.00 µl	135.2 µl

   b) Dispense 15 µl of the PCR reaction into 8 separate PCR tubes, add 10 µl of DNA extracted from individual monoclonal cell lines (Section D, Step 9).
   c) PCR amplify the target sequences in a Thermocycler using the parameters in Step 3b.
   d) Separate 5 µl of the PCR reaction in 2% agarose gel as described in Step 3c and visualize.

5) Purify the PCR amplicons obtained from the wild type cells and monoclonal cell lines.
   We typically use a Wizard® SV Gel and PCR Clean-up System (Promega).
6) Sequence the PCR amplicons using the Forward screening primer (Step 2b).
   Note: We typically sequence PCR amplicons of 4 individual monoclonal cell lines for each of the 3 sgRNA for a single gene to obtain one monoclonal cell line per sgRNA with a null mutation.
   We typically sequence PCR amplicons using Genewiz (https://www.genewiz.com) by diluting the purified PCR amplicons to 4 ng/µl and combining 10 µl of diluted PCR amplicon with 5 µl of 5 µM Forward screening primer.
7) Analyze the sequences using the Inference of CRISPR Edits (ICE) tool [2].
   The following three sequences are required for sequence analysis using ICE tool:
   - The 20 nucleotide forward sgRNA sequence (Section A, Step 6).
   - The sequence file of the wild type target site generated using the Forward sequencing primer (Step 6).
   - The sequence file of the monoclonal cell line target site generated using Forward screening primer (Step 6).

   a) Go to the ICE tool at https://ice.synthego.com/#/.
   b) Paste the 20 nucleotide sgRNA sequence in the box designated 'Guide sequences'.
   c) Drop or upload the .ab1 sequence file of the wild type target site to the box designated 'Control File'.
   d) Drop or upload the .ab1 sequence file of monoclonal cell line target site to the box designated 'Experiment File'.
   e) Analyzing the sequence. Identify monoclonal cell lines that contain indels in both chromosomes and that are predicted to result in frameshifts, preferably that lead to premature truncation of the protein. Typically, more than 50% of the monoclonal cell lines will contain such mutations. If not, screen additional candidates (Step 6) by sending their PCR amplicons for sequencing.
8) Expand monoclonal cell lines from Section D, Step 10 as described in Section F below.
   Note: Unless there is a duplicate stock plate of the candidate monoclonal cell lines stored at -80°C (Section D, Step 10), it is recommended to thaw the cells only once sufficient isolates with null mutations for each sgRNA target sites have been identified (Steps 4-7).

F. Expansion of CRISPR-Cas9 edited monoclonal cell lines.

1) For each monoclonal cell line to be expanded, aliquot 3 ml of DMEM + 10% FBS pre-warmed to 37°C into the wells of a 6-well tissue culture-treated plate.
2) For monoclonal cells to be expanded from growing cell culture (Section D, Step 10):
   a) Aspirate spent medium from culture, add 50 µl of 0.05% trypsin-EDTA and incubate the plate at 37°C for 5 min or until the cells begin to lift off the plate.
   b) Add 150 µl of DMEM + 10% FBS and resuspend the cells by pipetting.
   c) Centrifuge the cells at 234 ×*g* for 5 min at RT.
   d) Aspirate the medium and re-suspend the cells in 200 µl of DMEM + 10% FBS.
   e) Transfer the cell suspension to a designated well in the 6-well plate (Step 1).
   f) Incubate the cells at 37°C until they are 70-80% confluent.

   For monoclonal cells to be expanded from frozen stock at -80°C (Section D, Step 10):
   a) Thaw the 96-well plate of monoclonal cell lines in a 37°C water bath.
   b) Transfer the desired monoclonal cell lines to their designated wells in the 6-well tissue culture treated plate (Step 1).
   c) Centrifuge the plate at 234 ×*g* for 5 minutes at RT.
   d) Aspirate the medium and replace it with 3 ml of pre-warmed DMEM + 10% FBS.
   e) Incubate the cells at 37°C until they are 70-80% confluent.

3) Expand individual monoclonal cell lines.
   a) Re-suspend the cells in culture medium by scraping and transfer the cell suspension to a 15 ml conical tube.
   b) Centrifuge the cells at 234 ×*g* for 5 min at RT.
   c) Aspirate the medium and re-suspend the cells in 10 ml of DMEM + 10% FBS.
   d) Transfer the cells to 10 cm tissue culture-treated Petri dish (or T175 flask) and incubate at 37°C until the cells are 70-80% confluent (typically 2-3 days).

4) Harvest the cells as described in Step 3 re-suspending in 10 ml of DMEM + 10% FBS and determine the cell concentration using a hemocytometer. A single plate typically yields 1.5×10^6 cells/ml.
5) For cryo-preservation, dilute the cells to 7.5×10^5 cell/ml in 6 ml of DMEM + 10% FBS containing 10% DMSO, aliquot 6× 1 ml volumes in cryopreservation tubes and freeze overnight at -80°C. The following day transfer the tubes to the liquid nitrogen for long-term storage. Use the remaining cells for expansion (Step 6).
6) To prepare whole cell lysates to test for the target gene expression by Western analysis (Section G), dilute cells 1:10 in 15 ml DMEM + 10% FBS and culture in a 10 cm tissue culture-treated Petri plate (or T175 flask) at 37°C for 2 days.

G. Verification of target protein depletion by Western analysis.

1) Harvest the cells by scraping in spent medium and transfer the cell suspension to a 15 ml conical tube.
2) Centrifuge the cells at 234 ×*g* for 5 min at RT.
3) Aspirate the medium and resuspend the cells in 1.5-2.0 ml of DMEM + 10% FBS.
4) Dilute cells 1:20 by combining 50 µl of the cell suspension with 950 µl DMEM + 10% FBS in an Eppendorf tube and mix to homogeneity.
5) Determine the cell concentration using a hemocytometer.
6) To prepare whole cell lysates:
   a) Transfer 1×10^7 cells to 1.5 ml Eppendorf tube.
   b) Pellet the cells by centrifuge at 90 ×*g* for 5 minutes at RT

c) Aspirate the medium and re-suspend the cells in 200 µl of 2×SDS-PAGE sample buffer [100 mM Tris-Cl (pH 6.8), 4% (w/v) sodium dodecyl sulfate, 0.2% (w/v) bromophenol blue, 20% (v/v) glycerol and 200 mM dithiothreitol].
d) Incubate the cells at 95°C for 10 min.
e) Lysates can be stored at -20°C.

7) For Western analysis, separate 2-20 µl of cell lysate sample by SDS-PAGE, transfer to nitro-cellulose (or PVDF) membrane and probe the membrane for the desired protein. Below are general guidelines but should be optimize based on the protein of interest and antibodies used.
a) Block the membrane with 5% non-fat milk in 1× PBS-T for 30 minutes at RT.
b) Incubate the membrane in blocking buffer containing primary antibody specific to the target protein overnight at 4°C.
c) Wash the membrane 3 times with 1×PBS-T, 5 min each at RT.
d) Incubate the membrane in blocking buffer containing horseradish peroxidase (HRP)-conjugated secondary antibody for 1-2 hours at RT.
e) Wash the membrane 4 times with 1×PBS-T, 5 min each at RT.
f) Incubate the membrane with Amersham ECL™ Start Western Detection Reagent (GE Healthcare) or equivalent for 1-2 min and image.

Strip the blot and re-probe for tubulin or an equivalent internal loading control.
g) Wash the membrane 4 times with 1×PBS-T, 5 min each at RT.
h) Incubate the membrane in 15 ml striping buffer [2% SDS, 62.5 mM Tris-HCl pH 6.8, 100 mM 2-mercaptoethanol] for 1 hr at 56°C.
i) Wash the membrane 6 times with 1×PBS-T, 5 min each at RT.
j) Incubate the membrane with Amersham ECL™ Start Western Detection Reagent (GE Healthcare) or equivalent for 1-2 min and image to verify that the antibodies have been stripped off completely.
k) Wash the membrane 4 times with 1×PBS-T, 5 min each at RT.
l) Block and probe the membrane with α-tubulin antibody (1:20,000-1:500,000) (Sigma, T5168), horseradish peroxidase (HRP)-conjugated secondary antibody and develop following Steps a-f.

References

1. Smale ST. Transfection by electroporation of RAW 264.7 macrophages. *Cold Spring Harb Protoc.* 2010.
2. Conant D, Hsiau T, Rossi N, Oki J, Maures T, Waite K, et al. Inference of CRISPR Edits from Sanger Trace Data. CRISPR J. 2022;5(1):123-30.
