# Supplementary material for: An efficient and cost-effective method for disrupting genes in RAW264.7 macrophages using CRISPR-Cas9
Source: PLoS One. 2024 Mar 14;19(3):e0299513. doi: 10.1371/journal.pone.0299513 (PMC10939251; doi:10.1371/journal.pone.0299513)
Supplement: S1 Table — (PDF) [file pone.0299513.s004.pdf]

**Table S1: Oligonucleotides**

| Name | Gene ID *Mus musculus* | Sequence[a] | Reference |
|---|---|---|---|
| EF63xnlsYFP2aCas9F | - | CTTCTCTCTGTTAAAGCAAGCAGGCGA CGTGGAAGAAAACCCCGGTCCTGGTC | This study |
| EF63xnlsYFP2aCas9R | - | GCCGCGTGGCTTCGGCCGTTTCCCCGGA GTCGAACAGGAGGGCGCCAATG | This study |
| sgRNAseqF | - | TGTCTCATGAGCGGATACATATTTGA | This study |
| sgRNAscaffold-F1 | - | CCGAAAAGTGCCACCTGACGTCCGGGT TTATTACAGGGACAGCAGAGATCCA | This study |
| sgRNAscaffold-R1 | - | GCAGCTGGTGGAGTTAGAACATTTG | This study |
| sgRNAscaffold-F2 | - | GTTCTAACTCCACCAGCTGCTGAGCGAT TCTATCACCCAAATCA | This study |
| sgRNAscaffold-R2 | - | ATCTCCCGATCCGTCGACGTCACTGACG GGCACCGGAGCCAATTCCCACTC | This study |
| cas9seqR | - | GATCAACCGCAAGTCAGCCTTATC | This study |
| YFP-cas9seqF | - | TGAGCGATTCTATCACCCAAATCA | This study |
| Negative Cont-F | | caccgGCACTACCAGAGCTAACTCA | 54 |
| Negative Cont-R | | aaacTGAGTTAGCTCTGGTAGTGCc | 54 |
| PMP70 #1F | 19299 | caccgGCTCACACGGTACCTCTACG | This study |
| PMP70 #1R | 19299 | aaacCGTAGAGGTACCGTGTGAGCc | This study |
| PMP70 #2F | 19299 | caccgAACCAGGTACCCGACGACAG | This study |
| PMP70 #2R | 19299 | aaacCTGTCGTCGGGTACCTGGTTc | This study |
| PMP70 #3F | 19299 | caccgGTGAAATGACTAGATTGGCT | This study |
| PMP70 #3R | 19299 | aaacAGCCAATCTAGTCATTTCACc | This study |
| PMP70-1scF | 19299 | CCATTCTGTACATGCTACTCCCTTCC | This study |
| PMP70-1scR | 19299 | CCGATTTGGGAGGATAAAAGAGAAGT | This study |
| PMP70-2scF | 19299 | ACTGTCCCTACAGGCATGAATTTTGT | This study |
| PMP70-2scR | 19299 | GTTTCAAAGCACTGTTTTCAGCCTGT | This study |
| Pex19 #1F | 19298 | caccgACAGCACATCCTTAGACAGG | This study |
| Pex19 #1R | 19298 | aaacCCTGTCTAAGGATGTGCTGTc | This study |
| Pex19 #2F | 19298 | caccgGATGGTCGGAGCATGTTCTG | This study |
| Pex19 #2R | 19298 | aaacCAGAACATGCTCCGACCATCc | This study |
| Pex19 #3F | 19298 | caccgGCTGGCTTCCCAAGCTACTG | This study |
| Pex19 #3R | 19298 | aaacCAGTAGCTTGGGAAGCCAGCc | This study |
| Pex19#1seqFn | 19298 | GTTCTTTCCACCGGAAAAAGTGGTA | This study |
| Pex19#1seqRn | 19298 | TTTCCCCTTTTAGCTCAAACATTCC | This study |
| Pex19 #2seqF | 19298 | CACTGGTTCTGTGTAAAAGGGTTTCC | This study |
| Pex19 #2seqR | 19298 | AAGCCAGCTCACTGTCAAACAGTTC | This study |
| Pex19#3seqF | 19298 | CTTGATGATTTCGACAAAGCCAAAC | This study |
| Pex19#3seqR | 19298 | TAAGCCACTACACCTGGCTGGAATA | This study |
| PEX5 #1F | 19305 | caccgCTGGACTCACCATCGATCAG | This study |
| PEX5 #1R | 19305 | aaacCTGATCGATGGTGAGTCCAGc | This study |
| PEX5 #2F | 19305 | caccgTCGTGCGGCAGATTGGCGAG | This study |
| PEX5 #2R | 19305 | aaacCTCGCCAATCTGCCGCACGAc | This study |
| PEX5 #3F | 19305 | caccgTCTTGTAAACTGATCAACCC | This study |
| PEX5 #3R | 19305 | aaacGGGTTGATCAGTTTACAAGAc | This study |
| Pex5#1seqF | 19305 | TCTGAAGAGAACATTGGGTCTCCTG | This study |
| Pex5#1seqR | 19305 | GCCTTCAAGACCTTGAGGAACTACG | This study |
| Pex5#2seqF | 19305 | GGGTGATTTGGTTTTTCTTTCTTGC | This study |

| | | | |
|---|---|---|---|
| Pex5#2seqR | 19305 | CTGAAGGGCAGCTATTTTGTTTCCT | This study |
| Pex5#3seqFn2 | 19305 | TCCCACTGTCACAAAGATAGCCTTC | This study |
| Pex5#3seqRn2 | 19305 | CCCTGACTCTACCCCAGATTCTGAT | This study |
| PEX10 #1F | 668173 | caccgGTACGTTGGGATCATCCAAG | This study |
| PEX10 #1R | 668173 | aaacCTTGGATGATCCCAACGTACc | This study |
| PEX10 #2F | 668173 | caccgAAGGACGAGTACTACCTGGG | This study |
| PEX10 #2R | 668173 | aaacCCCAGGTAGTACTCGTCCTTc | This study |
| PEX10 #3F | 668173 | caccgACCTGGCCAAGAGACTAGCA | This study |
| PEX10 #3R | 668173 | aaacTGCTAGTCTCTTGGCCAGGTc | This study |
| Pex10#1seqF | 668173 | GACAGCGTCAGGTAAGCAGTGGTAT | This study |
| Pex10#1seqR | 668173 | CTCCGCTCTTTCCCAAGTACAGAAT | This study |
| Pex10#2seqF | 668173 | GTACCTCCTGCCAGAAAACTGAACC | This study |
| Pex10#2seqR | 668173 | GTCACTCCTAAGACGACCCTCAACA | This study |
| GNPAT #1F | 14712 | caccgCCTTCGGCTTAGGAACTCCG | This study |
| GNPAT #1R | 14712 | aaacCGGAGTTCCTAAGCCGAAGGc | This study |
| GNPAT #2F | 14712 | caccgGTGATCCCCCTATACAAGGG | This study |
| GNPAT #2R | 14712 | aaacCCCTTGTATAGGGGGATCACc | This study |
| GNPAT #3F | 14712 | caccgATGAGCCACAAACTGCGCAT | This study |
| GNPAT #3R | 14712 | aaacATGCGCAGTTTGTGGCTCATc | This study |
| GNPAT#1seqF | 14712 | ATTCATACACGGCGTGATTTGTTTC | This study |
| GNPAT#1seqR | 14712 | CCTTGGCACCAAGTTATACGTGTTC | This study |
| GNPAT#2seqF | 14712 | CTCTGGTCCAACAAAACAAAACCAC | This study |
| GNPAT#2seqR | 14712 | GGAAAGCCTGGATCTTTGTCAGGTA | This study |
| GNPAT#3seqF | 14712 | TGGGTTAATGTGTTTGGTTGCTAGG | This study |
| GNPAT#3seqR | 14712 | ATCTGCATGAGCACAGAGAAAATCC | This study |
| Pex7 #1F | 18634 | caccgAGTCTATAAAGAGCACACGC | This study |
| Pex7 #1R | 18634 | aaacGCGTGTGCTCTTTATAGACTc | This study |
| Pex7 #2F | 18634 | caccgATGGGATCAAACTGTCAAAG | This study |
| Pex7 #2R | 18634 | aaacCTTTGACAGTTTGATCCCATc | This study |
| Pex7 #3F | 18634 | caccgAGGCTGAAGCGAAACAACCA | This study |
| Pex7 #3R | 18634 | aaacTGGTTGTTTCGCTTCAGCCTc | This study |
| Pex7#1seqF | 18634 | TGAGTGTTTGGAAACCTCCTTTCAG | This study |
| Pex7#1seqR | 18634 | AGTTAGGCATGCATACTGCTCTTGC | This study |
| Pex7#2seqF | 18634 | AGGGAGCAAGCTTTTCAAGATGACT | This study |
| Pex7#2seqR | 18634 | TAAATGACACTCTCATGGCCCCTAA | This study |
| Pex7#3seqFn | 18634 | CAAAAGTGTTTCTGTTTACCTTTGTTGA | This study |
| Pex7#3seqRn | 18634 | GACAGGCAGGGAAAGAAAGAAAGAA | This study |
| PEXRAP #1F | 216820 | caccgAATGCTGGGATTAGCTACCG | This study |
| PEXRAP #1R | 216820 | aaacCGGTAGCTAATCCCAGCATTc | This study |
| PEXRAP #2F | 216820 | caccgCAATAGTACCAGGATCCGCG | This study |
| PEXRAP #2R | 216820 | aaacCGCGGATCCTGGTACTATTGc | This study |
| PEXRAP #3F | 216820 | caccgCCTTCACATTTCGTCCACAG | This study |
| PEXRAP #3R | 216820 | aaacCTGTGGACGAAATGTGAAGGc | This study |
| PEXRAP#1seqF | 216820 | ACACCATATATGATCCCACCCTGTG | This study |
| PEXRAP#1seqR | 216820 | AGGGTGGTTTGTTGGAAAGAAGAGA | This study |
| PEXRAP#3seqFn | 216820 | GTGCAGGGTTGAGTGACTTTGAACT | This study |
| PEXRAP#3seqRn | 216820 | GCGGACAATAGCAATATAGGGGGTA | This study |

[a] Lowercase nucleotides indicate overhangs that generate *Bsm*HI compatible cohesive ends for cloning annealed oligonucleotides into pYFP:Cas9-sgRNA.
