# Supplementary material for: An efficient and cost-effective method for disrupting genes in RAW264.7 macrophages using CRISPR-Cas9
Source: PLoS One. 2024 Mar 14;19(3):e0299513. doi: 10.1371/journal.pone.0299513 (PMC10939251; doi:10.1371/journal.pone.0299513)
Supplement: S2 Table — (PDF) [file pone.0299513.s005.pdf]

Table S2: Plasmids

Plasmid ID	Plasmid Name	Description	Reference
pTO1396	pEF6-nls-YFP-2A-Cas9	CRISPR-Cas9 delivery vector with single guide RNA (sgRNA) scaffold containing gBlock	30
pTO1406	pYFP:Cas9v1	pEF6-3xnls-YFP-2A-Cas9 lacking the 3.3 kb *Aat*II fragment	This study
pTO1417	pYFP:Cas9v2	pYFP:Cas9v1 with the two *BsmB*I sites in the Cas9 gene inactivated	This study
pTO1463	pCRISPR-EASY	CRISPR-Cas9 and sgRNA delivery vector	This study
pTO1480	pCRISPR-EASY::PMP70#1	sgRNA-1 delivery vector *Pmp70*	This study
pTO1482	pCRISPR-EASY::PMP70#2	sgRNA-2 delivery vector *Pmp70*	This study
pTO1484	pCRISPR-EASY::PMP70#3	sgRNA-3 delivery vector *Pmp70*	This study
pTO1486	pCRISPR-EASY::Non-target-sgRNA	non-target sgRNA control delivery vector	This study
pTO1599	pCRISPR-EASY::PEX5#1	*Pex5* sgRNA-1 delivery vector	This study
pTO1601	pCRISPR-EASY::PEX5#2	*Pex5* sgRNA-2 delivery vector	This study
pTO1603	pCRISPR-EASY::PEX5#3	*Pex5* sgRNA-3 delivery vector	This study
pTO1605	pCRISPR-EASY::PEX10#1	*Pex10* sgRNA-1 delivery vector	This study
pTO1607	pCRISPR-EASY::PEX10#2	*Pex10* sgRNA-2 delivery vector	This study
pTO1609	pCRISPR-EASY::PEX10#3	*Pex10* sgRNA-3 delivery vector	This study
pTO1611	pCRISPR-EASY::GNPAT#1	*Gnpat* sgRNA-1 delivery vector	This study
pTO1613	pCRISPR-EASY::GNPAT#2	*Gnpat* sgRNA-2 delivery vector	This study
pTO1615	pCRISPR-EASY::GNPAT#3	*Gnpat* sgRNA-3 delivery vector	This study
pTO1617	pCRISPR-EASY::PEXRAP#1	*Dhrs7b* (encoding PexRAP) sgRNA-1 delivery vector	This study
pTO1619	pCRISPR-EASY::PEXRAP#2	*Dhrs7b* sgRNA-2 delivery vector	This study
pTO1621	pCRISPR-EASY::PEXRAP#3	*Dhrs7b* sgRNA-3 delivery vector	This study
pTO1623	pCRISPR-EASY:PEX7#1	*Pex7* sgRNA-1 delivery vector	This study
pTO1625	pCRISPR-EASY::PEX7#2	*Pex7* sgRNA-2 delivery vector	This study
pTO1627	pCRISPR-EASY::PEX7#3	*Pex7* sgRNA-3 delivery vector	This study
pTO1629	pCRISPR-EASY::PEX19#1	*Pex19* sgRNA-1 delivery vector	This study
pTO1631	pCRISPR-EASY::PEX19#2	*Pex19* sgRNA-2 delivery vector	This study
pTO1633	pCRISPR-EASY::PEX19#3	*Pex19* sgRNA-3 delivery vector	This study
